# Prediction model for unsuccessful return to work after hospital-based intervention in low back pain patients

**DOI:** 10.1186/1471-2474-14-140

**Published:** 2013-04-19

**Authors:** Ole Kudsk Jensen, Kristian Stengaard-Pedersen, Chris Jensen, Claus Vinther Nielsen

**Affiliations:** 1 The Spine Center, Diagnostic Center, Silkeborg Regional Hospital, Silkeborg, Denmark; 2 Department of Rheumatology, Aarhus University Hospital, Aarhus, Denmark; 3 Department of Clinical Social Medicine, Institute of Public Health, University of Aarhus, Aarhus, Denmark; 4 National Centre for Occupational Rehabilitation, Rauland, Norway

**Keywords:** Low back pain, Prediction model, Range of motion, Radiculopathy, Return to work, Lumbar disc herniation, Psychological distress, Validation

## Abstract

**Background:**

Many studies on low back pain (LBP) have identified prognostic factors, but prediction models for use in secondary health care are not available. The purpose of this cohort study, based on a randomised clinical study, was to identify risk factors for unsuccessful return to work (U-RTW) in sick-listed LBP patients with or without radiculopathy and to validate a prediction model for U-RTW.

**Methods:**

325 sick-listed LBP patients with or without radiculopathy were included in an intervention study and followed for one year. Afterwards, 117 other LBP patients were recruited similarly, included in a validation study and also followed for one year. All patients were subjected to identical procedures and interventions and received a brief intervention by the same rehabilitation doctor and physiotherapist. Half of them received case manager guidance within a multidisciplinary setting. At baseline, they completed a questionnaire and went through a clinical low-back examination. Sciatica was investigated by magnetic resonance imaging (MRI). U-RTW was registered in a national database both initially and at 1-year.

**Results:**

Neither initial U-RTW (24.0%) nor one-year U-RTW (38.2%) were statistically significantly different in the two intervention groups nor in patients with and without radiculopathy. Multivariate logistic regression analysis identified two clinical and five psychosocial baseline predictors for one-year U-RTW (primary outcome). The clinical predictors included pain score (back+leg pain) and side-flexion. The five psychosocial predictors included ‘bodily distress’ ‘low expectations of RTW’, ‘blaming the work for pain’, ‘no home ownership’ and ‘drinking alcohol less than once/month’. These predictors were not statistically significantly different in patients with and without radiculopathy, and they also predicted initial U-RTW (secondary outcome). Obesity and older age were only supplementary predictors in patients with radiculopathy. A prediction model was established and tested in the validation study group. The model predicted one-year U-RWT in patients with intermediate and high risk, but only partially in patients with low risk. The model predicted all three risk categories in initial U-RTW.

**Conclusions:**

A prediction model combining baseline clinical and psychosocial risk factors predicted patients with low, intermediate and high risk for unsuccessful return to work, both initially and at 1-year.

## Background

Risk factors for sick-listed low back pain (LBP) patients’ unsuccessful return to work (U-RTW) have been a focus area in LBP research for many years due to the high costs of sick-listing [1-4]. In primary care, prediction models for continued pain, disability and sickness absence have been developed and validated [5]. The use of a simple screening questionnaire [6] has made it possible to target intervention at specific subgroups and thereby improve their overall outcome, including sick-listing [7]. However, no prediction models have been successfully validated in secondary care of LBP patients [8].

Pain-related risk factors have been identified in second care of LBP patients, e.g. pain intensity, leg pain, pain duration, high disability and widespread pain [9,10]. Neurologic findings and restriction of the spinal range of motion have been recognized as risk factors in some studies, but psychosocial risk factors were found to be more important [11,12]. Psychosocial risk factors may include psychological distress, especially somatisation or depression [13], negative expectations about RTW [4], low job satisfaction [2], no availability of modified job function [14], the belief that work is the only cause of the pain [15], low income [12] and even low alcohol intake [16,17]. In systematic reviews only a limited number of negative prognostic factors has been consistently identified as risk factors for non-specific LBP outcome [18]. The interplay between clinical and psychosocial risk factors, however, remains insufficiently clarified.

Most studies on risk factors have only included non-specific LBP with or without leg pain, but psychosocial factors have also been shown to affect RTW in patients with radiculopathy [1,19,20]. Whether psychosocial factors are more or less important in patients with radiculopathy than in patients with non-specific LBP remains unknown.

RTW may be defined as working continuously for four weeks during the first year [21], working full-time at one year [22], or not being full-time sick-listed at one year [14]. Different measures may produce different rates of RTW which may help explain the different associations found across studies.

In the present study, initial RTW was defined as working full-time at least 4 weeks continuously within the first year, and one-year RTW was defined as working full-time at least 4 weeks up to the one-year date after inclusion. Patients registered as unemployed, but otherwise fit to work, were also considered returned to work. All other patients were considered to be U-RTW. Furthermore, patients with radiculopathy were also included.

The aims of the present study were 1) to study the associations between baseline variables and U-RTW including clinical, psychosocial and life style aspects comparing risk factors in patients with and without radiculopathy; and 2) to establish and validate a prediction model for U-RTW with one-year U-RTW as primary outcome and initial U-RTW as secondary outcome.

## Methods

### Design

A prospective cohort study based on a randomized intervention study with one-year follow-up followed by a similar validation study with one-year follow-up.

### Patients

*The original study group.* During the period 2004-2007, general practitioners (GPs) referred 351 patients to the Research Unit of the Spine Centre. Information about the project was communicated to 163 GPs by meetings and letters. Each GP referred a mean of two (range 1-14) patients. At the first visit, all patients received a brief intervention (BI) by a rehabilitation specialist and a physiotherapist and half were randomized for supplementary multidisciplinary intervention (MDI). The interventions have been described in detail elsewhere [23]. In short, all patients had a BI lasting 2-3 hours by a rehabilitation doctor (OKJ) and a physiotherapist. For all patients, a follow-up visit was scheduled at the physiotherapist two weeks later, and a follow-up visit was arranged at the physician for patients needing answers in relation to their test results. In the BI group, the subsequent medical care was managed by the GP who also offered advice on RTW. For patients in the MDI group, a visit was scheduled for an interview with a case manager within two to three working days after the first consultation. The patient and the case manager together made a tailored rehabilitation plan that aimed at full or partial RTW.

Inclusion criteria: Partly or fully sick-listed from work for 4-12 weeks due to LBP with or without radiculopathy, LBP should be the prime reason for sick-listing and at least as bothersome as any possible pain elsewhere, age 16-60 years, referred from a well-defined area counting about 280,000 inhabitants, and the patient should be able to speak and understand Danish.

Exclusion criteria: Registered as unemployed, living outside the referral area, continuing or progressive radiculopathy resulting in plans for surgery, low back surgery within the past year, previous lumbar fusion operation, suspected cauda equina syndrome, progressive paresis or other serious back disease, (e.g. tumour), pregnancy, known dependency on drugs or alcohol or primary psychiatric disease.

Twenty-six of the 351 randomized patients were excluded for the following reasons: metastatic malignancy of the spine (2), osteoporotic fractures (4), spondylolisthesis (6), osteomalacia (2), peripheral arteriosclerosis (2), sacroiliitis (1), severe scoliosis (1), hydronephrosis (1), trochanteric bursitis (1), withdrawing after inclusion (4), 61 years old (1), sudden death during follow-up (1). This left 325 patients for the present study. Self-reported sick-listing was median 41 days, (mean 46 days, range 3-16 weeks). A total of 24 patients were sick-listed for less or more than 4-12 weeks. Eight patients arrived 1-6 days before the four-week date of sick-listing and their consultation could not be postponed. Sixteen patients had their first consultation postponed because of summer holidays.

*The validation study group.* After recruitment to the original study had closed, a second 12-month study was conducted with identical procedures and interventions, including the same randomization as in the original study. This group was used to test the applicability of the prediction model constructed from the original study. In total, 120 patients were included and followed for one year until July 2009. Three patients were excluded due to spondylolisthesis (2) and psoriatic arthritis (1), which left 117 patients for the validation project, 68 women and 49 men. They were sick-listed for median 44 days (range 25-85 days).

### Baseline variables

At inclusion, all patients completed a comprehensive questionnaire showing the low back shaded from the inferior costal margins to the gluteal folds. The questionnaire included items from both validated [5,24-30] and non-validated instruments. These questions as well as the physical examination and imaging are described in Table 1.

**Table 1:** Characteristics of baseline variables

**Data source**	**Categories of measure**
**Questionnaire**	
*Age*	Years
*Sex*	Female, male
*Pain intensity (Low Back Bain Rating Scale*[27]*)*	Sum of pain now (0-10), average pain (0-10) and worst pain (0-10) during the preceding 2 weeks
Back pain intensity	Numeric Rating Scale (0-30)
Leg pain intensity	Numeric Rating Scale (0-30)
Back+leg pain intensity (pain score)	Numeric Rating Scale (0-60), the sum of back and leg pain intensity
*Two additional questions*	Does leg pain spread to the lower part 1) of the leg 2) of the foot?
*Duration of actual pain*	Less than 3 months, 3-6 months, 6-12 months, ≥ 1 year
*Use of pain medication*	5-7 days per week, 1-4 days per week, 0 days
*Functional level (Roland Morris Questionnaire, validated Danish translation, 23 items*[28]*)*	Questions about limitations of daily activities because of LBP
	(0-23), increases when disabilities of daily activities increases
*Psychological distress, 4 subscales (Common Mental Disorders Questionnaire, CMDQ*[29]*)*	Bothered by the symptom during the past four weeks: ‘0’ if not bothered at all, ‘1’ if bothered a little, moderately, quite a bit or extremely. The subscales were calculated by adding the answers of each item.
Bodily distress	11 questions: ‘Headaches?’, ‘Dizziness or faintness?’, ‘Pains in heart or chest?’, ‘Nausea or upset stomach?’, ‘Soreness of your muscles?’, ‘Trouble getting your breath?’, ‘Hot or cold spells?’, ‘Numbness or tingling in parts of your body?’, ‘A lump in your throat?’, ‘Feeling weak in parts of your body?’, ‘Heavy feelings in your arms and legs?’. One question of LBP was omitted
Worrying and health anxiety	7 questions like for example about worries, that there is something seriously wrong with the body, many different kinds of pain and aches, thoughts that the doctor might be wrong if telling not to worry, worries about the health, etc.
Mental distress	8 questions like for example about nervousness or shakiness inside, spells of terror or panic, feeling fearful and feeling that everything is an effort, etc.
Depressive symptoms	6 questions about feeling blue, feeling of worthlessness, thoughts of ending ones life, feelings of been trapped or caught, feeling lonely and blaming oneself for things
*Widespread pain (from the Danish*	Two questions covering the preceding two weeks:
*version of the General Health*	Much bothered by pain or discomfort in
*Questionnaire)*	1) neck, shoulders, arms, hands?
	2) back, buttocks, legs, knees and feet?
*Fear avoidance*[5]	3 questions (0-10) about physical activity causing increasing pain, increasing pain indicating stop of the activity, and lack of ability to do normal activity and work with present pain. Sum score.
*Work-related questions*	1) Blaming the work for LBP (work the only cause vs. partly or not the cause)
	2) Expectations about return to work within 6 months (10 box scale, 8-10 vs. <8)
	3) Ongoing compensation (compensation claim, yes or no).
*General health questions (Danish version of SF-36*[30]*)*	In general, how do you perceive your health: Splendid? Very good? Good? Not so good? Bad?
*Items of social aspects. Questions from a*	
*Danish Public Health Questionnaire*^*1*^	
School education	4 ordered categories
Vocational education	5 ordered categories
Job function	5 ordered categories, leader yes, no
Marital status	5 categories
Children	Yes, no
+/- home ownership	Yes, no
Personal and family income	4 ordered categories
Sports or exercise activity in leisure time	2 categories
Smoking	Never, previous, current
Alcohol habits	Frequency, 6 ordered categories
**Physical examination**	
Body Mass Index (BMI)	Kg/m^2^
Signs of nerve root compression	At least one of the following: Positive Lasegue ≤ 60º, missing or inhibited reflex, altered sensation in a dermatome or paresis.
Forward flexion	Modified Schober: Lumbosacral junction marked, a mark placed 10 cm more proximally and 5 cm more distally. The increment under forward bending measured.
Side-flexion	A mark set on the lateral side of the thigh where the fingertips end.
	A new mark set after maximal side-bending, the difference measured. Side -flexion computed as the mean of the right and left side.
Waddell’s signs	One or more of 8 signs: LBP worsened by axial loading or simulated
	rotation, significant change of Lasegue in the sitting position, diffuse sensory changes, tenderness by superficial palpation, moaning, holding the hands on the back, using walking aids.
Tender points. (*A standardised, validated examination method*[24]*)*	A gradually increasing pressure applied by the thumb at 18 spots on the body, the pressure increased up to 4 kg during 4 seconds. The spots located symmetrically on the neck, shoulders, forearms, second ribs, buttocks and legs. The pressure first demonstrated to the patient distally on the forearm, and the patient instructed to distinguish a firm pressure from pain. Only painful points counted as positive.
**Imaging**	
*X-rays of the lumbar spine *(*Validated method*[25,26]*)*	Disc height reductions measured on plain lateral X-ray by one of the authors (OKJ), classified as no height reduction: 0, 0-25% ≈ slight: 1, 25-75% ≈ moderate: 2 and ≥ 75% reduction ≈ severe: 3.
	Disc degeneration score L1-4: The sum of L1 through L4 scores.
	(Validation in 60 patients by blinded reevaluation of images: The agreement good or acceptable in the upper 4 segments (agreement 83-95%, Kappa 0.46-0.71), not at the lumbosacral segment (agreement 73%, Kappa 0.34). The sum score only comprised the sum of the 4 upper lumbar segments (agreement for the sum 68%, Kappa 0.54))
*Magnetic resonance imaging (MRI)*	T_1 _and T_2 _-weighted sequences. STIR sequences of the sacroiliac joints if
*Most examinations performed at Silkeborg Regional Hospital using a 0.7 T machine*	inflammatory back disease was suspected clinically.
	No standard grading system was applied. The images described by a specialist of radiology. All examinations evaluated by one of the authors as well (OKJ). When in doubt, the images were discussed with the back surgeons at weekly conferences.
**History, physical examination, MRI and questionnaire**	
*Low back pain (LBP) classification*	1) Non-specific LBP:
	*without* pain below the knee: 96 patients (30%), including 12 patientswith disc herniation without radiating pain or neurologic signs.
	*with* pain below the knee: 118 patients (36%), including 15 patients with disc herniation with referred pain to leg or foot, but no neurologic signs
	2) Radiculopathy:
	111 patients (34%) with radiating pain and signs of nerve root
	compression and disc herniation (n=97) or spinal stenosis (n=14). The symptomatic disc herniation was located at L5-S1 in 62 patients, L4-5 in 33 patients and L2-4 in 2 patients. Spinal stenosis was located laterally in 9 patients and centrally in 5 patients.

^1^Centre of Public Health, University of Aarhus, Denmark.

The patients were classified as having non-specific LBP or radiculopathy on the basis of symptoms, physical examination and magnetic resonance imaging (MRI) findings (Table 1). All patients with radicular symptoms or ‘red flags’ were examined by MRI of the lumbar spine. In the beginning, MRI was only performed on clinical indication resulting in MRI in half of the patients, but MRI was performed in all patients during the last year of inclusion and in the whole validation period. Based on questionnaire answers, the patients with non-specific LBP were subdivided into two groups: patients with or without pain below the knee (Table 1). In the original study group, MRI was performed in 64% of patients with non-specific LBP and in 98% of patients with radiculopathy.

Surgery: Patients with radiculopathy were referred for surgical evaluation if conservative therapy brought no improvement. In the original study group, 31 patients (9.5%) were operated, 9% in the BI and 10% in the MDI group (p=0.85). In the validation study group, 10 patients (8.5%) were operated with no statistically significant difference between the two intervention groups (p=0.195).

### Outcome variables

Initial RTW was defined as receiving no social transfer payments except for unemployment benefits continuously for 4 weeks during the first year after inclusion. Initial U-RTW included all other patients. One-year RTW was defined as receiving no social transfer payments except for unemployment benefits during the last 4 weeks up to the one-year-date after inclusion. One-year U-RTW included all other patients and was defined as the primary outcome. Patients were identified in a national database [31] that registers all social transfer payments on a weekly basis. Follow-up was therefore 100%. Social transfer payments comprise compensation benefits for unemployment, sick-listing, job-training, further education, supported job-function and disability pension. Sick-listing for less than two weeks is compensated by the employer and is not registered in this database.

### Ethical approval

The trial was discussed with the regional research ethics committee. Approval was not necessary, because all patients received the best available clinical care and no biological material was involved. The study was approved by the Danish Data Protection Agency (No. 2007-41-1278). All patients signed informed consent.

### Analyses

Differences in RTW between the two intervention groups were analysed by Chi^2^-test, and the t-test was used for comparing baseline pain scores and degrees of side-flexion in the two study groups.

The reliability of the classification of disc degeneration ascertained on X-rays was analysed by Kappa statistics (one observer, two observations, non-weighted, Table 1).

Univariate analyses in the original study group were performed by logistic regression with adjustment for age and sex, first with one-year U-RTW as outcome, afterwards with initial U-RTW as outcome. Furthermore, there was analysed for interaction (effect modification) between patients with and without radiculopathy. Discrete numerical and ordered categorical variables were analyzed for linearity by logodds plots. Subsequently, multivariate analysis was performed by first analysing clinical variables and establishing a clinical multivariate model. Well-known risk factors were included first, marked as ‘^w’^ in Table 2. Potential risk factors, marked as ^‘p’^ in Table 2, and all other risk factors were included in turn. Categorical variables were checked by Wald’s test. Collinearity was checked by multiple correlation analysis. A variable was kept in the model if p≤0.05 and excluded if p>0.05, except if this was caused by collinearity. If collinearity between one and another variable was suspected, change of estimates, confidence limits and p-values were evaluated when both variables were included in the model as compared to one variable included. If collinearity was confirmed by this procedure, either the variable in question was excluded from the model if p>0.05 or combined with the other variable. When including a new variable, previously excluded variables were tentatively included again. Effect modification was incorporated into the model. Furthermore, the model was checked by Hosmer and Lemeshow´s goodness-of-fit test. Afterwards, a psychosocial multivariate model was established in a similar way.

**Table 2:** Baseline variables and logistic regression analyses of univariables

		**One-year U-RTW **^**1**^		
**Variables**	**Baseline**	**OR**	**95% CI**	**P**
*Clinical variables*				
Sex: female/all (% female), reference female	166/325 (51)	0.74	0.47-1.66	0.196
Age^w^: mean (SD, range), reference 18 years	41.7 (10.4, 18-60)	1.00	0.98-1.02	0.743
Body Mass Index (BMI): mean (SD, range), ref. 18 kg/m^2^	26.7 (5.0, 18-53)	1.01	0.97-1.06	0.597
N^o^ with radiculopathy/all, n (%), ref. non-specific	111/325 (34)	1.06	0.65-1.72	0.829
Low back pain classification, n (%) overall p				0.011
Non-specific LBP without radiation below the knee	96 (30)	1		
Non-specific LBP with pain below the knee^w^	118 (36)	2.19	1.21-3.97	
Radiculopathy^w^	111 (34)	1.87	1.00-3.50	
Intensity of back pain^2p^, mean (SD, range), ref. 0	17.6 (6.3, 0-30)	1.12	1.08-1.17	<0.001
Intensity of leg pain^2^, mean (SD, range), ref. 0	14.2 (8.3, 0-30)	1.06	1.03-1.09	<0.001
Pain score^2p^ (back + leg pain), mean (SD, range), ref. 3	32.0 (12.2, 3-60)	1.06	1.04-1.08	<0.001
Duration of actual pain^p^: ref. ≤ 3 months (%)	(51)	1.35	0.85-2.13	0.200
Use of pain medication: (%5-7 days/week), ref. less often	(58)	1.75	1.09-2.80	0.021
Disability (Roland Morris)^w^: median (range), ref. 3	16 (3-23)	1.19	1.03-1.16	0.003
N^o^ ‘much bothered by widespread pain^p^ the preceding two weeks’/all, n				
(%), reference: ‘not much bothered’	53/325 (16)	1.43	0.77-2.66	0.254
Forward-flexion (Mod. Schober): mean (SD, range), ref. 0.5 cm	5.3 (1.6, 0.5-10)	0.96	0.83-1.11	0.574
Side-flexion^p^: mean (SD, range), reference 4 cm	13.7 (3.8, 4-26.5)	0.89	0.83-0.95	<0.001
Tender points^p^: median (range), reference 0	5 (0-18)	1.07	1.01-1.13	0.017
Disc Degenerat. Score L1-4: 0-12, median (range), ref. 0	1 (0-8)	0.95	0.80-1.12	0.521
Disc herniation^p^ L4-5 without radiculopathy, ref. none	12/325 (4)	0.86^3^	0.24-3.04	0.818
Disc herniation^p^ L5-S1 without radiculopathy, ref. none	14/325 (4)	1.55^3^	0.52-4.61	0.427
*Psychosocial and life style variables*				
Fear avoidance^w^: (0-30), dichotomised, reference <28	25 (3-30)	1.62	1.27-2.06	<0.001
Waddell’s signs^w^: n/all (% with one or more), ref. none	78/325 (24)	2.33	1.37-3.97	0.002
Bodily distress^w^: 0-11, median (range), ref. 0	3 (0-11)	1.19	1.08-1.30	<0.001
Worrying and health anxiety^w^: 0-7, median (range), ref. 0	2 (0-7)	1.13	1.01-1.27	0.039
Mental distress^w^: 0-8, median (range), ref. 0	1 (0-8)	1.13	1.03-1.24	0.011
Depressive symptom^w^: 0-6, median (range), ref. 0	0 (0-6)	1.23	1.08-1.40	0.002
General health perceived as bad^w^/all, reference: splendid, very good, good				
or not so good	33/322 (10)	3.25	1.53-6.89	0.002
N^o^ ‘blaming work for low back pain^p^’/ all, n (%), ref. ‘not blaming work				
or only partly blaming work’	70/315 (22)	2.40	1.40-4.12	0.002
N^o^ ‘not convinced about return to work within 6 months^w^’/all, n (%), ref.				
‘convinced about return to work’	119/323 (37)	3.69	2.27-6.00	<0.001
N^o^ with compensation claim^w^/all, n (%), ref. no claim	77/316 (24)	2.16	1.27-3.65	0.004
School education^p^, n (%) overall p				0.353
< 10 years	99 (31)	1		
10 years	120 (37)	0.69	0.40-1.21	
high school or alike	70 (22)	0.60	0.31-1.18	
something else	33 (10)	0.55	0.23-1.28	
Vocational education, n (%) overall p				0.611
none	55 (17)	1		
unskilled, one or more courses	41 (13)	1.12	0.49-2.59	
skilled education, craftsman, clerk	110 (35)	0.74	0.38-1.45	
short and intermediate education < 4 years	81 (25)	0.67	0.33-1.37	
long education > 4 years	8 (3)	0.38	0.07-2.07	
Marital status overall p				0.085
married	158 (49)	1		
living together, not married	85 (27)	1.26	0.72-2.23	
alone, not previously living together	12 (4)	0.17	0.02-1.38	
alone (previous married or living together)	48 (15)	1.41	0.72-2.73	
something else	16 (5)	3.10	1.05-9.16	
N^o^ with no children/all, n (%), ref. having children	71/320 (22)	0.91	0.50-1.66	0.755
N^o^ with no home ownership/all, n (%), ref. home ownership	108/318 (34)	2.61	1.60-4.27	<0.001
Job^w^ overall p				0.978
unskilled	104 (33)	1		
skilled	73 (23)	1.11	0.60-2.07	
salaried employee	66 (21)	1.00	0.51-1.95	
independent	17 (5)	0.76	0.25-2.34	
something else	56 (18)	1.04	0.52-2.06	
Job: N^o^ leader/all, n (%), ref. not leader	37/308 (12)	0.49	0.22-1.10	0.085
Personal income^p^ €, n (%) overall p				0.019
<20,137	33 (11)	1		
20,137-33,561	153 (50)	0.57	0.26-1.24	
33,562-50,341	99 (32)	0.34	0.14-0.79	
>50,341	22 (7)	0.18	0.05-0.67	
Income of family €, n (%) overall p				0.278
< 33,562	46 (16)	1		
33,562-50,341	70 (25)	1.52	0.71-3.26	
50,342-67,123	106 (37)	0.91	0.44-1.87	
> 67,123	63 (22)	0.48	0.21-1.11	
Smoking^p^, n (%) overall p				0.065
never smoking	101 (31)	1		
previously smoking	85 (26)	1.41	0.76-2.63	
smoking currently	136 (42)	1.93	1.11-3.36	
N^o^ drinking alcohol less than once per month^p^/all, n (%), ref. drinking				
regularly, at least once/month	77/322 (24)	1.76	1.03-2.98	0.037
Exercise in leisure time^p^, dichotomised				
Vigorous or regular exercise several times a week including heavy				
gardening or housework, n (%)	108 (34)	1		
Walking, cycling or light exercise some hours a week or no exercise at				
all, n (%)	212 (66)	1.27	0.78-2.06	0.340

Outcome: unsuccessful return to work at one year (one-year U-RTW). The analyses adjusted for age and sex, except age and sex.

^1 ^Not succeeding in working for at least 4 weeks up to the one-year date or registered as unemployed for at least 4 weeks up to the one-year date: 124 patients (38.2%).

^2 ^Transformed to VAS scale 0-10: mean back pain 5.9, mean leg pain 4.7 and mean pain score 5.3.

^3 ^Patients not examined by MRI were excluded as the diagnosis requires MRI of the lumbar spine.

^w ^Well-known risk factor. ^p ^Potential risk factor.

OR: Odds Ratio. CI: confidence interval.

The clinical and psychosocial models were combined by including first one variable from one model, afterwards one variable from the other model, then again another variable from the first model and so on. The same principles were used for keeping and excluding variables and handling collinearity as described above.

Multivariate models for initial U-RTW, which was less frequent than one-year U-RTW, were analyzed and established in a similar way, but fewer variables could be included (maximum of 10 observations per variable).

The statistical package STATA[32] was used, and a significance level of 5%, two-sided, was chosen.

## Results

### The original study group

Initial RTW was registered in 247 patients (76.0%). The remaining 78 patients (24.0%) were registered as initial U-RTW with no statistically significant difference between the two intervention groups: BI 22.3%, MDI 25.8% (p=0.46).

At one-year follow-up, 58.1% were registered as receiving no social transfer payments, and 3.7% were registered as receiving unemployment benefits. RTW was considered successful in these 201 patients (61.8%). One-year U-RTW included the remaining 38.2% (124 patients, 69 women and 55 men). There was no statistically significant difference between the two intervention groups: BI 35.5%, MDI 40.9% (p=0.32). Seventy-eight of these patients (62.9%) were full-time or part-time sick-listed. The others were in job-training, under further education, retired or had a supported job-function or were receiving disability pension.

Accordingly, 46 patients (18.6% of 247) who were initially registered as RTW were registered as U-RTW at one year.

About one third of the patients had radiculopathy; two thirds had non-specific LBP, half of whom had pain below the knee. Men more often had radiculopathy than women (61 vs. 39%, p=0.001).

### The validation study group

Initial RTW was registered in 81 patients (69.2%). The remaining 36 patients (30.8%) were registered as initial U-RTW with no statistically significant difference between the two intervention groups: BI 32.8%, MDI 28.8% (p=0.64).

At one-year follow-up, 49.6% were registered as receiving no social transfer payments, and 3.4% were registered as receiving unemployment benefits. RTW was considered successful in these 62 patients (53.0%). One-year U-RTW included the remaining 47.0% (55 patients, 35 women and 20 men). There was no statistically significant difference between the two intervention groups: BI 50.0%, MDI 44.1% (p=0.52).

Accordingly, 19 patients (23.4% of 81) who were initially registered as RTW were registered as U-RTW at one year.

Clinically, 46 patients (39%) had radiculopathy, which in all patients except one was verified by MRI. One-year U-RTW did not differ between patients with radiculopathy (39%) and patients without (45%), p=0.526. Radiculopathy tended to occur more often in men than in women (47% vs. 34%), p=0.152.

### Clinical baseline variables - univariate analyses

The ‘Disability of daily activities’ scores were high (median 16 of 23) as were the pain scores (back+leg pain), mean 32.0 of 60 (5.3 on a 0-10 scale). Half of the patients reported pain lasting for more than three months (Table 2).

No statistically significant differences in one-year U-RTW were observed between patients with and without radiculopathy, but patients with non-specific pain above the knee had lower risk for one-year U-RTW than those with pain below the knee, whether the pain was non-specific or due to radiculopathy (Table 2).

Clinical risk factors associated with one-year U-RTW were the pain scores, use of pain medicine, disability, tender points and side-flexion.

Psychosocial variables associated with one-year U-RTW were ‘fear avoidance’, ‘non-organic signs’, all four types of psychological distress, ‘perceiving general health as bad’, ‘blaming the work for pain’, ‘compensation claim’, ‘low expectations of RTW’, ‘low personal income’, ‘no home ownership’ and ‘drinking alcohol less than once/month’ (Table 2).

Univariate analyses with initial U-RTW as outcome are shown in the Additional file 1: Table S1).

### Multivariate analyses

The results of the initial multivariate analyses are only shown in the Additional file.

The clinical model included the pain score and side-flexion and furthermore age and body mass index (BMI) in patients with radiculopathy (effect modification). Disability of daily activities’ did not contribute to the model because of collinearity with the pain score (Additional file 1: Table S2).

The psychosocial model included ‘Bodily distress’, ‘low expectations of RTW’, ‘blaming the work for pain’, ‘drinking alcohol less than once/month’ and ‘fear avoidance’.

**Figure 1:**
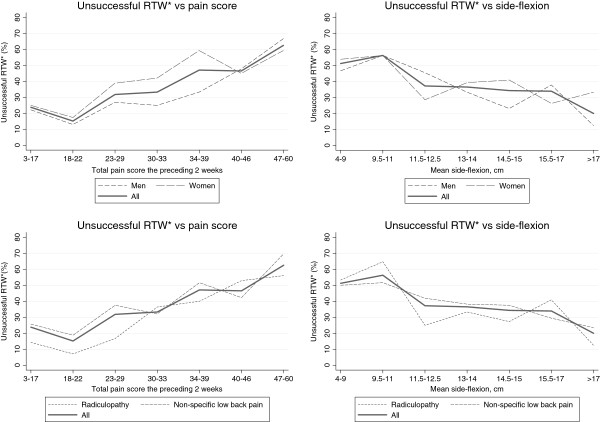
Percentage with unsuccessful return to work (*RTW) at one year in relation to the pain score (back+leg pain) and side-flexion. The upper panel shows men and women, the lower panel shows patients with and without radiculopathy.

The combined clinical and psychosocial model included the pain score and side-flexion, the ‘bodily distress’ variable and 4 dichotomous psychosocial variables: ‘low expectations of RTW’, ‘blaming the work for pain’, ‘no home ownership’ and ‘drinking alcohol less than once/month’ (Additional file 1: Table S2). The pain score and side-flexion were combined to a combination variable. Both variables were normally distributed and were mutually inversely associated with one-year U-RTW as illustrated by Figure 1. In order to create groups with different risk levels based on clinical factors, these two variables were combined in the following way:

The two variables were dichotomized by using the medians as cut points. The resulting four groups were combined as follows: one low-pain-good-motion group, one low-pain-restricted-motion group, one high-pain-good-motion group and one high-pain-restricted-motion group. The two intermediate groups were combined into one group because they did not differ significantly in terms of risk of one-year U-RTW (p=0.165). Thus, the resulting pain/side-flexion variable had three categories with different risk profiles in relation to one-year U-RTW (Additional file 1: Table S2 and Additional file 1: Table S3).

A final combination variable was constructed to create three equally sized risk groups with as different risk profiles as possible. The candidate variables for the prediction model were the remaining variables from the combined model with one-year U-RTW as outcome: ‘bodily distress’ and the 4 dichotomous variables ‘low expectations of RTW’, ‘blaming the work for pain’, ‘no home ownership’ and ‘drinking alcohol less than once/month’. These variables were combined with the pain/side-flexion variable as shown in Additional file 2: Table S3. The process is further illustrated in the Additional file 1: Table S3 which also shows the logistic regression analysis of the combination variable of the 4 dichotomous risk factors. The OR´s of the four dichotomous risk factors varied from 1.93 to 3.75 (Additional file 1: Table S3) which only corresponded to a small difference in U-RTW of 22% and 30%, respectively. The four risk factors were therefore combined as if they contributed equally.

### The final prediction model

The final model was used for predicting both one-year U-RTW and initial U-RTW (Table 3). By using the final combination variable, all combinations of clinical information (pain/side-flexion variable) and psychosocial information were tabulated in relation to risk groups (Table 4). Although the model was derived from the pain/side-flexion model, some of the patients in the ‘low’ risk group had high levels of pain and restricted side-flexion (pain/side-flexion group 3), and some of the patients in the ‘high’ risk group had little pain and no restriction of side-flexion (pain/side-flexion group 1).

**Table 3:** Multivariate logistic regression analyses resulting in 2 final prediction models

**Variables**	**OR**	**95% CI**	**P**
***One-year U-RTW, final prediction model, N=282***			
Risk groups: Final combination variable Overall p			<0.001
(pain/side-flexion, bodily distress, 4 dichotomous variables^2^)			
Low	1		
Intermediate	5.04	2.11-12.02	
High	15.5	6.56-36.41	
Radiculopathy^1^, ref. non-specific LBP	0.57	0.27-1.120	0.138
BMI in non-spec. LBP group, ref. 25 kg/m^2^	0.97	0.91-1.03	0.305
Effect modification BMI:			
OR(BMI radiculopathy)/OR(BMI non-specific)	1.33	1.12-1.57	0.001
Age in non-spec. LBP group, ref. 40 years	0.99	0.96-1.03	0.589
Effect modification age:			
OR(age radiculopathy)/ OR(age non-specific)	1.08	1.01-1.15	0.025
*AUC 0.79.*			
*Cut point 0.38: 71% correctly classified*			
***Initial U-RTW, final model, N=282***			
Risk groups: Final combination variable Overall p			<0.001
(pain/side-flexion, bodily distress, 4 dichotomous variables^2^)			
Low	1		
Intermediate	3.23	1.24-8.40	
High	7.94	3.20-19.7	
Radiculopathy^1^, ref. non-specific LBP	0.82	0.39-1.74	0.611
BMI in non-spec. LBP group, ref. 25 kg/m^2^	0.99	0.92-1.06	0.695
Effect modification BMI:			
OR(BMI radiculopathy)/OR(BMI non-specific)	1.24	1.06-1.44	0.006
Age	1.03	1.00-1.06	0.082
*AUC 0.73*			
*Cut point 0.25: 66% correctly classified*			

The upper model with unsuccessful return to work at one year as outcome (one-year U-RTW).

The lower model with unsuccessful return to work during the year as outcome (initial U-RTW).

Adjustment for intervention group did not change the models. There was no effect modification for the final combination variable between patients with and without radiculopathy (one-year U-RTW, p=0.253, initial U-RTW, p=0.842).

^1 ^Interpreted as the difference of return to work in two patients with and without radiculopathy, both persons 40 years old with BMI 25 kg/m^2^ and not different regarding other risk factors.

^2 ^Final combination variable of pain/side-flexion (3 groups), bodily distress (4 groups) and 4 dichotomous risk factors: ‘Not convinced about return to work within 6 months’, ‘blaming the work for pain’, ‘drinking alcohol less than once/month’, ‘no home owner ship’.

43 missing values in the combination model: Pain score (9), bodily distress (19), blaming the work for pain (7), home ownership (4), drinking alcohol less than once/month (3), BMI (1).

OR: Odds Ratio. CI: Confidence interval. AUC: Area under curve

**Table 4:** Combinations of pain/side-flexion, ‘4 risk factors’ and ‘bodily distress’ in three risk groups

***Low risk***			***Intermediate risk***			***High risk***		
**Pain/side-flexion Group**	**N**^**o **^**of 4 risk factors**	**N**^**o **^**of bodily distress symptoms**	**Pain/side-flexion Group**	**N**^**o **^**of 4 risk factors**	**N**^**o **^**of bodily distress symptoms**	**Pain/side-flexion Group**	**N**^**o **^**of 4 risk factors**	**N**^**o **^**of bodily distress symptoms**
1	0	0-11	1	1	6-11	1	2	5-11
1	1	0-5	1	2	0-4	1	3	0-11
2	0	0-7	2	0	8-11	2	1	8-11
2	1	0-2	2	1	3-7	2	2	3-11
3	0	0-4	2	2	1-2	2	3	0-11
			3	0	5-11	3	1	5-11
			3	1	0-4	3	2	0-11
						3	3	0-11
						3	4	0-11

Pain/side-flexion group:

Group 1 (low-pain-good-motion): Back+leg pain ≤32 & side-flexion ≥13.5 cm

Group 2 (intermediate group): Back+leg pain ≤32 & side-flexion <13.5 cm

*or *back+leg pain >32 & side-flexion ≥13.5 cm

Group 3 (high-pain-restricted-motion): Back+leg pain >32 & side-flexion <13.5 cm

Number of risk factors (0-4):

‘Not convinced about return to work within 6 months’, ‘blaming the work for pain’, ‘drinking alcohol less than once/month’, ‘no home owner ship’.

Number of bodily distress symptoms the last 4 weeks (0-11).

‘Headaches?’, ‘Dizziness or faintness?’, ‘Pains in heart or chest?’, ‘Nausea or upset stomach?’, ‘Soreness of your muscles?’, ‘Trouble getting your breath?’, ‘Hot or cold spells?’, ‘Numbness or tingling in parts of your body?’, ‘A lump in your throat?’, ‘Feeling weak in

parts of your body?’, ‘Heavy feelings in your arms and legs?’

A combination variable with initial U-RTW as outcome developed from the combination variable also shown in Table 3, was not used, because it was no better than the final prediction model at predicting initial U-RTW, and it predicted one-year U-RTW poorly.

### Effect modification and adjustment – one-year U-RTW

There was no statistically significant effect modification between the patients with and without radiculopathy for any of the seven predictors included in the final model (not shown), or when analysing the final prediction model (Table 3). The estimates of the seven predictors did not vary when adjusted for intervention group or when the analyses were restricted to patients who were examined by MRI (analyses not shown).

### Validation of the prediction model

Expected point estimates of risks with 95% confidence intervals for both U-RTW outcomes were calculated from the two logistic regression analyses in the original study group shown in Table 3. The final combination variable was defined separately in the dataset of the original study group and the validation study group. Afterwards, the observed numbers of patients with one-year U-RTW and initial U-RTW in both study groups were tabulated by use of the final combination variable (Table 5).

**Table 5:** Validation of prediction model

***Risk category***	***Low***	***Intermediate***	***High***
***Original study group***			
**N=282 n (% of N)**	91 (32)	86 (31)	105 (37)
**Risk for ‘initial U-RTW’**^**1 **^**predicted by final model *% (95% CI)***	*7.2 (3.2-15.3)*	*19.3 (11.8-29.3)*	*34.9 (25.2-46.1)*
**Observed number: obn obn/n**	7/91	18/86	39/105
**Observed risk %**	*7.7*	*20.9*	*37.1*
**Risk for ‘one-year U-RTW’**^**2**^	*9.1 (4.3-18.1)*	*33.5 (23.4-45.3)*	*60.6 (49.3-71.0)*
**predicted by final model *% (95% CI)***			
**Observed number: obn obn/n**	9/91	29/86	62/105
**Observed risk %**	*10.2*	*33.7*	*59.1*
***Validation study group***			
**N=104 n (% of N)**	27 (26)	26 (25)	51 (49)
**‘Initial U-RTW’**^**1**^**, observed number: obn obn/n**	3/27	5/26	20/51
**Observed risk %**	*11.1*	*19.2*	*39.2*
**‘One-year U-RTW’**^**2**^**, observed number obn/n**	7/27	7/19	30/59
**Observed risk %**	*25.9*	*38.5*	*58.8*

Upper panel: Predicted risks with 95% confidence intervals for unsuccessful return to work (U-RTW) and corresponding observed risks in the original study group.

Lower panel: Observed risks for U-RTW in the validation study group.

^1 ^Not succeeding in working continuously for at least 4 weeks work during the first year after inclusion or registered as unemployed for at least 4 weeks.

^2 ^Not succeeding in working continuously for at least 4 weeks up to the one-year date or registered as unemployed for at least 4 weeks up to the one-year date.

The figures apply for patients with non-specific LBP and patients with radiculopathy who were middle aged (~40 years) and with BMI ~25. Predicted risks for patients with radiculopathy, who were older and/or obese were higher (not shown because of few patients in subgroups, see article text “Small subgroups..”).

CI: Confidence interval.

Expected risks for initial U-RTW were generally lower than for one-year U-RTW, and there were overlap between the three confidence intervals (Table 5). Expected risks in regard to one-year U-RTW were differentiated into three groups with no overlap between confidence intervals. Observed risks for initial U-RTW were in good accordance with expected risks in both the original study group and the validation study group.

In the original study group, observed risk for one-year U-RTW was also in good accordance with expected risks.

In the validation study group, observed risks for one-year U-RTW were within the confidence intervals of the ‘intermediate’ and ‘high’ risk group, but was located above the upper confidence limit of the ‘low risk’ group. The observed ‘low’ risk was still lower than the observed ‘intermediate’ risk (25.9% vs. 38.5%).

In the validation group, the patients reported more total pain, and side-flexion was more restricted than in the original study group: pain score mean 35.25 vs. 31.96 (p=0.014, t-test), side-flexion mean 12.09 vs. 13.66 (p<0.001, t-test). The other predictors were not statistically significantly different in the two study groups.

### Small subgroups with increased risk of U-RTW

Due to effect modification, the observed risk for one-year U-RTW in the original study group was high in obese patients with radiculopathy (U-RTW=65.2%, BMI>30, n=23), and it was also high in older patients with radiculopathy (U-RTW=52.4%, age>55, n=21). In the validation study group, the corresponding figures were 41.7% (BMI>30, n=12) and 71.4% (age>55, n=9).

Missing patients in the model (42=15%) had moderately elevated risk for one-year U-RTW. Observed risk for this group was 54.8% and 31.0% for the two U-RTW outcomes, respectively.

## Discussion

Our prediction model could predict the primary outcome, one-year U-RTW, in ‘intermediate’ and ‘high’ risk patients, but with less precision in ‘low’ risk patients. The ‘intermediate’ and ‘high’ risk patients were reliably differentiated from each other, as there was no overlap between the confidence intervals, and the observed numbers were within the confidence limits. The model also predicted initial U-RTW (secondary outcome) well in all three risk groups as the observed numbers were within the confidence limits. Because of overlap between the confidence intervals, only ‘low’ and ‘high’ risk patients could be reliably differentiated from each other in initial U-RTW. The prediction model may be easy to use since only few measures are required to classify a patient: one clinically measured variable (side-flexion) and 6 questionnaire-based items. In addition, BMI and age may have to be considered in patients with radiculopathy.

We believe that this prediction model for secondary-health-care LBP patients is better than the prognostic evaluation used at present. For instance, a back surgeon often consider the results of MRI of the lumbar spine more important than other aspects, a rehabilitation doctor may give priority to ‘yellow flags’ and a specialist of social medicine may focus primarily on social risk factors.

Approximately 20% of the patients registered as initial RTW shifted to U-RTW at one-year follow-up probably indicating sickness relapse. We cannot exclude other reasons for relapse of sick-listing than LBP, but in the original study group most of these patients belonged to the ‘high’ risk group presumably being more at risk for relapse. The percentage relapse of sick-listing was well in accordance with a previous study [22].

Apart from BMI and age, the predictors only included two clinical, four psychosocial and one life style measure. The other variables associated with U-RTW in the univariate analyses did not contribute to the final model, although some are well-known from other studies to be risk factors. This was especially true for ‘fear avoidance’, ‘disability of daily activities’, ‘leg pain’ [19], ‘widespread pain’[17], ‘non-organic signs’[33] and ‘perceived poor general health’. This should not be interpreted as if these other risk factors are not important, but they simply did not contribute as predictors in the model.

In the original study group the percentage with initial RTW was 76%, and the percentage with one-year RTW was 61.8% which corroborates previous studies [22,34,35]. RTW was lower in the validation study group (53.0%), maybe because this group suffered from more pain and had more restricted side-flexion than the original study group; but it may also be explained by external factors. We hypothesize that an alternative explanation may be the financial crisis, which also hit Denmark during the spring of 2009.

### Disability

The disability level of the patients was high as reflected by a RMQ score of mean 15.6 as compared to a mean of 14.4 in the high risk group in the study of Hill et al. using the STarT Back tool to differentiate low, intermediate and high risk [7]. Thus, the patients were not average primary-care-patients, but selected high risk patients. We assume that the high disability was due to selection bias by GPs who were probably inclined to refer suspected high risk patients to secondary health care. The selection bias may also be due to more than 3-4 weeks sick-listing which is associated with increased risk in LBP patients [1]. The normal distribution of pain and side-flexion suggested a systematic bias, and most of the patients of the present study would probably have belonged to the STarT Back high-risk-group.

### Pain score

In other studies [14], a ‘high level of disability’ has been identified as a very significant risk factor. We also found a strong association between a ‘high level of disability’ and U-RTW in the univariate analyses. In the multivariate analysis, however, disability did not contribute because of collinearity with the pain score [36]. We believe that the LBP Rating Scale [27] yields a better pain registration than the pain registration used in many other studies. This scale reflects both the intensity of the pain and its location (back and/or leg pain) and covers the preceding 2 weeks. Moreover, in these patients the pain score was also associated with pain and function at one year [36].

### Side-flexion

Restriction of side-flexion has previously been identified as a risk factor for U-RTW [37,38]. Restriction of side-flexion may stem from increased muscle stiffness, which has been shown to result from muscle adaptation to pain [39]. It was easily measured like forward flexion (Modified Schober), but forward flexion was not associated with U-RTW.

### Psychosocial risk factors

Psychological distress is one of the best documented risk factors for adverse outcome in LBP patients [13,14,18]. The questionnaire used in the present study has been well validated [29] and has proven its value in a Danish context [40]. Many of the questions resemble questions of the General Health Questionnaire used in other LBP populations [17]. In the present study, especially the ‘bodily distress’ symptoms were able to predict outcome.

The first two of the four dichotomous predictors may be interpreted as cognitive risk factors: ‘low expectations of recovery’[10,14,15,41] and ‘blaming the work for pain’[41,42]. Noteworthy, ‘low expectations of recovery’ was the only risk factor identified consistently in a previous systematic review [4].

The predictor, ‘no home ownership’, has not previously been recognized as a risk factor for U-RTW, and this finding therefore has to be confirmed in other studies. However, it is important to bear in mind that the predictors identified do not necessarily represent causal relationships, but may be proxy markers for other risk factors, for instance social vulnerability or socio-economic status. The strength of this association did not change when adjusted for age.

The remaining predictor ‘drinking alcohol less than once/month’ was difficult to explain as a risk factor. However, a low intake of alcohol has been shown to be associated with pain in other studies [43,44], and also with outcome in LBP patients [16,17]. The association between pain conditions and low alcohol use might be rooted in other aspects than the purely biological effects of alcohol, for example social factors like previous overuse or parental overuse. Religious or cultural aspects may also be considered; yet, these hypotheses need to be confirmed in future studies.

### Older age and obesity in patients with radiculopathy

Older age is a well-established risk factor in non-specific LBP [19]; however, not in all studies [4]. In the present study, it was a predictor especially in patients with radiculopathy.

Obesity is a well-established risk factor in patients with clinically defined sciatica [45]. The dose-response relationship apparently present in this study may point to a mechanical mechanism, but cardiovascular explanations have also been hypothesized [45]. Obesity was not confirmed as a predictor in the validation study, but the subgroups were small. No final conclusion can therefore be drawn regarding this risk factor.

### Structural changes related to disc degeneration

Overall, patients with radiculopathy were facing the same risk of U-RTW as patients with non-specific LBP. The disc degeneration score L1-4 measured on X-ray was not associated with the prognosis. This is in accordance with other studies and has been confirmed in MRI studies [46-49]. The present study allows no conclusion about L5-S1 disc degeneration as measured on X-ray. In addition, no final conclusion can be drawn in regard to structural findings on MRI. However, in a coming paper we will show how vertebral endplate signal changes, so-called Modic changes, may affect the prognosis.

### Strengths

Sick-listing due to LBP was inclusion criteria in both patients with and without radiculopathy, and the referral from GPs to secondary health care makes the study resemble usual patient care.

Recruitment of the patients and the interventions were unchanged throughout the original and validation study period.

All patients were examined by the same experienced rheumatologist and physiotherapist.

RTW was registered by a national database with 100% follow-up.

### Limitations

The study was planned as a randomised controlled study, but was analyzed as a cohort study. However, there was no difference in RTW between the two intervention groups.

Examination by MRI of the lumbar spine was not performed in all patients, but the predictors were unchanged when the analyses were restricted to patients with MRI performed.

Subgroup analyses showed that the prediction model should be used with caution in patients with possibly increased risk of U-RTW like older or obese patients with radiculopathy.

The model should not be used in the patients with specific low back disorders, who were excluded from this study, or in patients treated with spinal surgery.

Work-place related risk factors have not been included in the present analysis, but analysis of these risk factors has been presented elsewhere [50]. Focus was not specifically heavy physical work which has been shown to be important in an inception cohort study [51]. Accordingly, the model should be used with caution in patients with heavy physical work.

The number of patients in the validation study group was limited, wherefore numbers in the subgroups were small; and missing values in some of the variables may have resulted in underestimation of the risk.

The estimates of the 4 dichotomous variables were different, and the combination variable was formed on the assumption that these risk factors were equally important. However, the maximal difference of estimates only corresponded to modest difference in risk for U-RTW (22% vs. 30%).

The present prediction model was solely based on baseline variables. Other circumstances within the first year after inclusion may have affected U-RTW such as differences in support by the social service centres of the municipalities, changes in the business cycle, surgery in a subset of patients or personal events.

### Perspectives

The prediction model may help explain why some patients with a low level of pain and no restriction of their range of motion have a high risk of U-RTW, i.e. because of the presence of many other risk factors, and why some patients with a high level of pain and a restricted range of motion may have a low risk of U-RTW, i.e. because no other risk factors are present. The model hence strengthens our understanding of the interplay between clinical and psychosocial risk factors. It may be hypothesized that the “disease” is LBP manifested as pain in the back/leg and/or restricted motion, which may be complicated by or elicited by structural changes in the spine. Its outcome in terms of U-RTW may then depend on the severity of the “disease” and any accompanying psychosocial risk factors.

Some of the predictors identified are not modifiable, for instance ‘older age, and ‘no home ownership’. Moreover, it is premature to recommend regular alcohol consumption, as the mechanism of this possible risk factor remains unclear. However, RTW may be positively affected by better low-back pain management (improving pain and motion). Furthermore, it is essential to improve care by focusing on interventions that may reduce psychological distress and by modifying the belief that work is the sole cause of pain as well as supporting self-confidence regarding RTW. Finally, weight reduction might improve RTW in patients with radiculopathy.

Identifying subgroups with different prognosis may enable the health care system to differentiate its care: Patients with a good prognosis may need only a brief intervention involving a rehabilitation doctor and physiotherapist, whereas other sick-listed patients may need a more extensive intervention.

We expect this preliminary prediction model to be useful in our setting, but more studies are needed to improve the model.

## Conclusions

The prediction model identified low, intermediate and high risk for initial unsuccessful RTW and also identified patients with high and intermediate risk for unsuccessful RTW at one year. Low risk at one year was predicted less precisely.

Both clinical and psychosocial predictors seemed to contribute to the risk for unsuccessful RTW. In the present study the predictors were not significantly different in patients with radiculopathy as compared to patients without, except for age and obesity affecting patients with radiculopathy more.

We advocate use of the prediction model for LBP patients referred to secondary health care because of sick-listing and receiving at least a brief intervention by a rehabilitation doctor and physiotherapist. The model should be used with caution in obese or older patients with radiculopathy and in patients with heavy physical work. Furthermore, the prediction model should not be used in patients with other specific back disorders or in patients treated by spinal surgery.

## Competing interests

The authors declare that they have no competing interests.

## Authors’ contributions

OKJ, KS and CVN planned the study. OKJ designed the study in detail and was responsible for clinical care and baseline data. CJ was responsible for acquisition of follow-up data and obtaining funding. OKJ was responsible for analysing and interpreting the data, and he was supervised by teachers at the Department of Biostatics, University of Aarhus. OKJ wrote the manuscript, which was again revised by KS, CVN and CJ. All authors discussed the results and commented on the manuscript. All authors read and approved the final manuscript.

## Pre-publication history

The pre-publication history for this paper can be accessed here:

http://www.biomedcentral.com/1471-2474/14/140/prepub

## Supplementary Material

Additional file 1: Table S1 Baseline variables and logistic regression analyses of univariables. **Table S2** Multivariate logistic regression analyses. **Table S3 **Logistic regression models with one-year U-RTW as outcome. Click here for file

Additional file 2: Table S3 Observed numbers of patients with one-year U-RTW^1 ^in three risk group combinations. Click here for file
