# Supplementary material for: Prediction model for unsuccessful return to work after hospital-based intervention in low back pain patients
Source: BMC Musculoskelet Disord. 2013 Apr 19;14:140. doi: 10.1186/1471-2474-14-140 (PMC3663778; doi:10.1186/1471-2474-14-140)
Supplement: Additional file 1: Table S1 — Baseline variables and logistic regression analyses of univariables. Table S2 Multivariate logistic regression analyses. Table S3 Logistic regression models with one-year U-RTW as outcome. [file 1471-2474-14-140-S1.doc]

**Table 1 (appendix) Baseline variables and logistic regression analyses of univariables.**

|  |  | **Initial U-RTW ^1^** | | |  | | **One-year U-RTW ^2^** | | |
| --- | --- | --- | --- | --- | --- | --- | --- | --- | --- |
| **Variables** | **Baseline** | **OR** | **95% CI** | **P** | | **OR** | | **95% CI** | **P** |
| *Clinical variables* |  |  |  |  | |  | |  |  |
| Sex: female/all (% female), reference female | 166/325 (51) | 0.92 | 0.56-1.54 | 0.763 | | 0.74 | | 0.47-1.66 | 0.196 |
| Age^w^: mean (SD, range), reference 18 years | 41.7 (10.4, 18-60) | 1.02 | 0.99-1.04 | 0.390 | | 1.00 | | 0.98-1.02 | 0.743 |
| Body Mass Index (BMI): mean (SD, range), ref. 18 kg/m^2^ | 26.7 (5.0, 18-53) | 1.03 | 0.98-1.08 | 0.282 | | 1.01 | | 0.97-1.06 | 0.597 |
| N^o^ with radiculopathy/all, n (%), ref. non-specific | 111/325 (34) | 1.37 | 0.79-2.36 | 0.257 | | 1.06 | | 0.65-1.72 | 0.829 |
| Low back pain classification, n (%) overall p  Non-specific LBP without radiation below the knee  Non-specific LBP with pain below the knee^w^  Radiculopathy^w^ | 96 (30)  118 (36)  111 (34) | 1  2.00  2.10 | 1.01-3.97  1.03-4.26 | 0.084 | | 1  2.19  1.87 | | 1.21-3.97  1.00-3.50 | 0.011 |
| Intensity of back pain^3p^, mean (SD, range), ref. 0 | 17.6 (6.3, 0-30) | 1.10 | 1.05-1.16 | <0.001 | | 1.12 | | 1.08-1.17 | <0.001 |
| Intensity of leg pain^3^, mean (SD, range), ref. 0 | 14.2 (8.3, 0-30) | 1.05 | 1.02-1.09 | 0.002 | | 1.06 | | 1.03-1.09 | <0.001 |
| Pain score^3p^ (back + leg pain), mean (SD, range), ref. 3 | 32.0 (12.2, 3-60) | 1.05 | 1.03-1.08 | <0.001 | | 1.06 | | 1.04-1.08 | <0.001 |
| Duration of actual pain^p^: ref. ≤ 3 months (%) | (51) | 1.34 | 0.80-2.25 | 0.270 | | 1.35 | | 0.85-2.13 | 0.200 |
| Use of pain medication: (%5-7 days/week), ref. less often | (58) | 1.35 | 1.03-1.78 | 0.032 | | 1.75 | | 1.09-2.80 | 0.021 |
| Disability (Roland Morris)^w^: median (range), ref. 3 | 16 (3-23) | 1.09 | 1.02-1.16 | 0.014 | | 1.19 | | 1.03-1.16 | 0.003 |
| N^o^ ‘much bothered by widespread pain^p^ the preceding two weeks’/all, n (%), reference: ‘not much bothered’ | 53/325 (16) | 1.20 | 0.60-2.43 | 0.604 | | 1.43 | | 0.77-2.66 | 0.254 |
| Forward-flexion (Mod. Schober): mean (SD, range), ref. 0.5 cm | 5.3 (1.6, 0.5-10) | 0.91 | 0.78-1.07 | 0.263 | | 0.96 | | 0.83-1.11 | 0.574 |
| Side-flexion^p^: mean (SD, range), reference 4 cm | 13.7 (3.8, 4-26.5) | 0.85 | 0.78-0.92 | <0.001 | | 0.89 | | 0.83-0.95 | <0.001 |
| Tender points^p^: median (range), reference 0 | 5 (0-18) | 1.06 | 1.00-1.13 | 0.042 | | 1.07 | | 1.01-1.13 | 0.017 |
| Disc Degenerat. Score L1-4: 0-12, median (range), ref. 0 | 1 (0-8) | 0.94 | 0.77-1.14 | 0.524 | | 0.95 | | 0.80-1.12 | 0.521 |
| Disc herniation^p^ L4-5 without radiculopathy, ref. none | 12/325 (4) | 1.49^4^ | 0.42-5.26 | 0.540 | | 0.86^4^ | | 0.24-3.04 | 0.818 |
| Disc herniation^p^ L5-S1 without radiculopathy, ref. none | 14/325 (4) | 2.06^4^ | 0.68-6.21 | 0.201 | | 1.55^4^ | | 0.52-4.61 | 0.427 |
| *Psychosocial and life style variables* |  |  |  |  | |  | |  |  |
| Fear avoidance^w^: (0-30), dichotomised, reference <28 | 25 (3-30) | 1.43 | 1.10-1.87 | 0.007 | | 1.62 | | 1.27-2.06 | <0.001 |
| Waddell’s signs^w^: n/all (% with one or more), ref. none | 78/325 (24) | 1.86 | 1.04-3.32 | 0.036 | | 2.33 | | 1.37-3.97 | 0.002 |
| Bodily distress^w^: 0-11, median (range), ref. 0 | 3 (0-11) | 1.14 | 1.03-1.26 | 0.013 | | 1.19 | | 1.08-1.30 | <0.001 |
| Worrying and health anxiety^w^: 0-7, median (range), ref. 0 | 2 (0-7) | 1.07 | 0.94-1.22 | 0.281 | | 1.13 | | 1.01-1.27 | 0.039 |
| Mental distress^w^: 0-8, median (range), ref. 0 | 1 (0-8) | 1.10 | 0.99-1.22 | 0.083 | | 1.13 | | 1.03-1.24 | 0.011 |
| Depressive symptom^w^: 0-6, median (range), ref. 0 | 0 (0-6) | 1.23 | 1.06-1.42 | 0.006 | | 1.23 | | 1.08-1.40 | 0.002 |
| General health perceived as bad^w^/all, reference: splendid, very good, good or not so good | 33/322 (10) | 2.69 | 1.27-5.68 | 0.010 | | 3.25 | | 1.53-6.89 | 0.002 |
| N^o^ ‘blaming work for low back pain^p^’/ all, n (%), ref. ‘not blaming work or only partly blaming work’ | 70/315 (22) | 2.37 | 1.33-4.22 | 0.004 | | 2.40 | | 1.40-4.12 | 0.002 |
| N^o^ ‘not convinced about return to work within 6 months^w^’/all, n (%), ref. ‘convinced about return to work’ | 119/323 (37) | 3.89 | 2.00-5.80 | <0.001 | | 3.69 | | 2.27-6.00 | <0.001 |
| N^o^ with compensation claim^w^/all, n (%), ref. no claim | 77/316 (24) | 2.01 | 1.14-3.56 | 0.016 | | 2.16 | | 1.27-3.65 | 0.004 |
| School education^p^, n (%) overall p  < 10 years  10 years  high school or alike  something else | 99 (31)  120 (37)  70 (22)  33 (10) | 1  0.46  0.43  0.68 | 0.25-0.87  0.20-0.93  0.27-1-67 | 0.063 | | 1  0.69  0.60  0.55 | | 0.40-1.21  0.31-1.18  0.23-1.28 | 0.353 |
| Vocational education, n (%) overall p  none  unskilled, one or more courses  skilled education, craftsman, clerk  short and intermediate education < 4 years  long education > 4 years | 55 (17)  41 (13)  110 (35)  81 (25)  8 (3) | 1  1.38  0.93  0.77  1.58 | 0.56-3.45  0.44-1.97  0.34-1.75  0.50-5.00 | 0.579 | | 1  1.12  0.74  0.67  0.38 | | 0.49-2.59  0.38-1.45  0.33-1.37  0.07-2.07 | 0.611 |
| Marital status overall p  married  living together, not married  alone, not previously living together  alone (previous married or living together)  something else | 158 (49)  85 (27)  12 (4)  48 (15)  16 (5) | 1  0.91  0.31  1.61  1.14 |  | 0.454 | | 1  1.26  0.17  1.41  3.10 | | 0.72-2.23  0.02-1.38 0.72-2.73  1.05-9.16 | 0.085 |
| N^o^ with no children/all, n (%), ref. having children | 71/320 (22) | 0.68 | 0.33-1.40 | 0.299 | | 0.91 | | 0.50-1.66 | 0.755 |
| N^o^ with no home ownership/all, n (%), ref. home ownership | 108/318 (34) | 2.01 | 1.17-3.46 | 0.011 | | 2.61 | | 1.60-4.27 | <0.001 |
| Job^w^ overall p  unskilled  skilled  salaried employee  independent  something else | 104 (33)  73 (23)  66 (21)  17 (5)  56 (18) | 1  1.40  0.92  1.40  1.02 | 0.70-2.79  0.43-1.99  0.44-4.44  0.46-2.25 | 0.805 | | 1  1.11  1.00  0.76  1.04 | | 0.60-2.07  0.51-1.95  0.25-2.34  0.52-2.06 | 0.978 |
| Job: N^o^ leader/all, n (%), ref. not leader | 37/308 (12) | 0.53 | 0.21-1.35 | 0.182 | | 0.49 | | 0.22-1.10 | 0.085 |
| Personal income^p^ €, n (%) overall p  <20,137  20,137-33,561  33,562-50,341  > 50,341 | 33 (11)  153 (50)  99 (32)  22 (7) | 1  0.72  0.54  0.32 | 0.31-1.67  0.21-1.38  0.07-1.37 | 0.378 | | 1  0.57  0.34  0.18 | | 0.26-1.24  0.14-0.79  0.05-0.67 | 0.019 |
| Income of family €, n (%) overall p  < 33,562  33,562-50,341  50,342-67,123  > 67,123 | 46 (16)  70 (25)  106 (37)  63 (22) | 1  1.42  1.14  0.72 | 0.59-3.42  0.49-2.64  0.27-1.88 | 0.449 | | 1  1.52  0.91  0.48 | | 0.71-3.26  0.44-1.87  0.21-1.11 | 0.278 |
| Smoking^p^, n (%) overall p  never smoking  previously smoking  smoking currently | 101 (31)  85 (26)  136 (42) | 1  1.45  1.66 | 0.72-2.94  0.88-3.12 | 0.291 | | 1  1.41  1.93 | | 0.76-2.63  1.11-3.36 | 0.065 |
| N^o^ drinking alcohol less than once per month^p^/all, n (%), ref. drinking regularly, at least once/month | 77/322 (24) | 1.66 | 0.93-2.98 | 0.087 | | 1.76 | | 1.03-2.98 | 0.037 |
| Exercise in leisure time^p^, dichotomised  Vigorous or regular exercise several times a week including heavy gardening or housework, n (%)  Walking, cycling or light exercise some hours a week or no exercise at all, n (%) | 108 (34)  212 (66) | 1  1.14 | 0.65-1.98 | 0.646 | | 1  1.27 | | 0.78-2.06 | 0.340 |

Two different outcomes: Initial unsuccessful return to work (initial U-RTW) and one-year U-RTW.

^1^ Not succeeding in working continuously for at least 4 weeks work during the first year after inclusion or registered as unemployed for at least 4 weeks: 78 patients (24.0%).

^2^ Not succeeding in working for at least 4 weeks up to the one-year date or registered as unemployed for at least 4 weeks up to the one-year date: 124 patients (38.2%).

The analyses adjusted for age and sex, except age and sex.

^3^ Transformed to VAS scale 0-10: mean back pain 5.9, mean leg pain 4.7 and mean pain score 5.3.

^4^ Patients not examined by MRI were excluded as the diagnosis requires MRI of the lumbar spine.

^w^ Well-known risk factor. ^p^ Potential risk factor.

OR: Odds Ratio. CI: confidence interval.

**Table 2 (appendix) Multivariate logistic regression analyses.**

| **Variables** | **OR** | **95% CI** | **p** |
| --- | --- | --- | --- |
| ***One-year U-RTW, clinical model, N=312*** |  |  |  |
| Pain score (back+leg pain), (3-60), ref. 3 | 1.06 | 1.03-1.08 | <0.001 |
| Side-flexion, (4-26 cm), ref. 4 cm | 0.92 | 0.86-0.99 | 0.027 |
| Radiculopathy^1^, ref. non-specific LBP | 0.48 | 0.24-0.89 | 0.020 |
| BMI in non-spec. LBP group, ref. 25 kg/m^2^ | 0.96 | 0.91-1.02 | 0.174 |
| Effect modification BMI:  OR(BMI radiculopathy)/OR(BMI non-specific) | 1.22 | 1.08-1.39 | 0.002 |
| *Adjusted for age and sex. AUC 0.73*  *Cut point 0.38, correctly classified 68%* |  |  |  |
| ***One-year U-RTW, psychosocial model, N=289*** |  |  |  |
| Bodily distress (0-11), ref. 0 | 1.18 | 1.06-1.31 | 0.002 |
| ‘Not convinced about return to work  Within 6 months’, ref. convinced | 2.90 | 1.65-5.09 | <0.001 |
| Blaming work for pain, ref. no or partial blame | 2.84 | 1.48-5.43 | 0.002 |
| Home ownership, ref. yes | 1.80 | 1.00-3.26 | 0.050 |
| Drinking alcohol less than once/month,  Ref. at least once/month | 2.18 | 1.12-4.22 | 0.021 |
| Fear avoidance, dichotomised, (0-30), ref. <28 | 1.43 | 1.08-1.90 | 0.014 |
| *Adjusted for age and sex. AUC 0.78*  *Cut point 0.38, correctly classified 70%* |  |  |  |
| ***One-year U-RTW, combined clinical and psychosocial model, N=282*** |  |  |  |
| Pain/side-flexion variable^2^ (group 1-3) Overall p  Group 1 (reference)  Group 2  Group 3 | 1  1.50  3.96 | 0.72-3.14  1.77-8.88 | 0.002 |
| Bodily distress (0-11), ref. 0 | 1.15 | 1.02-1.29 | 0.018 |
| ‘Not convinced about return to work  Within 6 months’, ref. convinced | 2.57 | 1.40-4.72 | 0.002 |
| Blaming work for pain, ref. no or partial blame | 3.75 | 1.86-7.57 | <0.001 |
| Home ownership, ref. yes | 1.93 | 1.03-3.63 | 0.040 |
| Drinking alcohol less than once/month,  Ref. at least once/month | 2.49 | 1.20-5.15 | 0.014 |
| Radiculopathy^1^, ref. non-specific LBP | 0.49 | 0.22-1.10 | 0.085 |
| BMI in non-spec. LBP group, ref. 25 kg/m^2^ | 0.97 | 0.91-1.04 | 0.359 |
| Effect modification BMI:  OR(BMI radiculopathy)/OR(BMI non-specific) | 1.31 | 1.11-1.54 | 0.002 |
| Age in non-spec. LBP group, ref. 40 years | 1.00 | 0.96-1.03 | 0.788 |
| Effect modification age:  OR(age radiculopathy)/ OR(age non-specific) | 1.08 | 1.01-1.16 | 0.024 |
| *Adjusted for sex. AUC 0.81*  *Cut point 0.38, correctly classified 73%* |  |  |  |
| ***Initial U-RTW, combined clinical and psychosocial model,***  ***N=285*** |  |  |  |
| Pain/side-flexion variable^2^ (group 1-3) Overall p Low | 1 |  | <0.001 |
| Intermediate | 0.88 | 0.38-2.04 |  |
| High | 5.00 | 2.00-10.1 |  |
| ‘Not convinced about return to work  Within 6 months’, ref. convinced | 2.95 | 1.56-5.59 | 0.001 |
| Blaming work for pain, ref. no or partial blame | 3.72 | 1.83-7.53 | <0.001 |
| Drinking alcohol less than once/month,  Ref. at least once/month | 2.53 | 1.201-5.28 | 0.014 |
| Radiculopathy1, ref. non-specific LBP | 0.70 | 0.32-1.52 | 0.359 |
| BMI in non-spec. LBP group, ref. 25 kg/m2 | 0.99 | 0.92-1.06 | 0.751 |
| Effect modification BMI:  OR(BMI radiculopathy)/OR(BMI non-specific) | 1.24 | 1.07-1.44 | 0.004 |
| Age in non-spec. LBP group, ref. 40 years | 1.04 | 1.01-1.07 | 0.016 |
| *Adjusted for sex. AUC 0.80*  *Cut point 0.25, correctly classified 73%* |  |  |  |

Primary outcome: 3 models with one-year U-RTW as outcome (upper three models).

Secondary outcome: 1 model with initial U-RTW* as outcome (lower model).

Adjustment for intervention group did not change the models.

^1^ Interpreted as the difference of return to work in two patients with and without radiculopathy, both persons 40 years old with BMI 25 kg/m^2^ and not different regarding other risk factors.

^2^ Combination variable of pain score and side-flexion: pain/side-flexion (3 groups).

OR: Odds Ratio. CI: Confidence interval. AUC: Area under curve

Not shown: The clinical model with initial U-RTW as outcome (almost identical with the clinical model with one-year U-RTW as outcome), and the corresponding psychosocial model (only differed by including ‘blaming the work for pain’ in stead of ‘fear avoidance’).

**Table 3 (appendix) Logistic regression models with one-year U-RTW as outcome.**

*The final combination variable in 3 steps:*

*Step1.* Pain/side-flexion:

n OR 95% CI p

Cat 1 88 1

Cat 2 115 2.14 1.11-4.13 0.023

Cat 3 80 5.37 2.69-10.7 <0.001

*Step 2.* Pain/side-flexion +

‘bodily distress’

n OR 95% CI p

Cat 1 64 1

Cat 2 131 2.37 1.10-5.13 0.028

Cat 3 88 7.44 3.63-16.5 <0.001

*Step 3.* Pain/side-flexion +

‘bodily distress’ +

‘4 risk factors’

n OR 95% CI p

Cat 1 91 1

Cat 2 86 4.64 2.04-10.53 <0.001

Cat 3 106 13.3 6.06-29.41 <0.001

The ‘4 risk factor’ model:

n OR 95% CI p

Cat 0 89 1

Cat 1 97 1.81 0.88-3.69 0.105

Cat 2 68 6.63 3.18-13.8 <0.001

Cat 3 25 10.5 3.83-28.7 <0.001

Upper three models showing the stepwise change after combining the models as described in the article (Table 3). ORs increased by every step. The total number included standardised to facilitate comparisons. The lower model showing logistic regression of the combination variable of the 4 risk factors (‘low expectations of RTW’, ‘blaming the work for pain’, ‘no home ownership’ and ‘drinking alcohol less than once/month’).

Cat: category, OR: odds ratio, 95% CI (confidence interval).
