# Supplementary material for: Prediction model for unsuccessful return to work after hospital-based intervention in low back pain patients
Source: BMC Musculoskelet Disord. 2013 Apr 19;14:140. doi: 10.1186/1471-2474-14-140 (PMC3663778; doi:10.1186/1471-2474-14-140)
Supplement: Additional file 2: Table S3 — Observed numbers of patients with one-year U-RTW1 in three risk group combinations. [file 1471-2474-14-140-S2.doc]

**Table 3 Observed numbers of patients with one-year U-RTW^1^** in three risk group combinations.

| **Original Study group**  **N= 283**  **Combination variables** | **Risk groups:** | **Low**    **% af N** | **Intermediate**    **% af N** | **High**    **% af N** |
| --- | --- | --- | --- | --- |
| **Pain/side-flexion** | Obs/n  % | 17/88 *31*  *19.3* | 39/115 *41*  *33.9* | 45/80 *28*  *56.3* |
| **Pain/side-flexion**  **plus ’bodily distress’** | Obs/n  % | 10/64 *23*  *15.6* | 40/131 *46*  *30.5* | 51/88 *31*  *58.0* |
| **Pain/side-flexion**  **plus ’bodily distress’ plus ‘4 risk factors’** | Obs/n  % | 9/91 *32*  *9.9* | 29/86 *30*  *33.7* | 63/106 *37*  *59.4* |

|  |  | Pain/side-flexion + ‘bodily distress’ | | |
| --- | --- | --- | --- | --- |
|  | Categ. | 1 | 2 | 3 |
| 4 psychosocial risk factors | 0 | 23 | 47 | 19 |
|  | 1 | 21 | 49 | 27 |
|  | 2 | 18 | 25 | 25 |
|  | 3 | 2 | 9 | 14 |
|  | 4 | 0 | 1 | 3 |

Low risk

| 91 |
| --- |
| 86 |
| 106 |

Intermediate risk

High risk

Upper panel showing what is achieved in differentiating the percentages of patients with U-RTW and making group sizes equal by combining three variables: The pain/side-flexion variable, the bodily distress variable and ‘the 4 risk factor variable’. The included number of patients was standardised to make comparisons meaningful. Percentages with U-RTW and percentages in each group shown with *Italics*.

*First step:* pain/side-flexion variable, a result of combining pain score with side-flexion, please se article text.

*Second step:* combining pain/side-flexion (3 categories) with ‘bodily distress’ (reduced to 4 equally sized categories) by adding the two variables to a combination variable (6 categories), this variable again reduced to 3 categories (1+2, 3+4, 5+6).

*Third step:* (lower panel) combining the pain/side-flexion-bodily-distress variable diagonally with a combination variable of the four risk factors resulting in a combination variable with 3 equally sized groups: the final prediction model.

^1^Not succeeding in working for at least 4 weeks up to the one-year date or registered as unemployed for at least 4 weeks up to the one-year date
